# Physician Satisfaction Following Electronic Health Record Adoption in Three Massachusetts Communities

**DOI:** 10.2196/ijmr.2064

**Published:** 2012-11-08

**Authors:** Leonie Heyworth, Fang Zhang, Chelsea A Jenter, Rachel Kell, Lynn A Volk, Micky Tripathi, David W Bates, Steven R Simon

**Affiliations:** 1 VA Boston Healthcare SystemSection of General Internal MedicineJamaica Plain, MAUnited States; 2 Brigham and Women's HospitalDivision of General Internal MedicineBoston, MAUnited States; 3 Department of Population Medicine, Harvard Medical School and Harvard Pilgrim Health Care InstituteBoston, MAUnited States; 4 Massachusetts eHealth CollaborativeWaltham, MAUnited States; 5 Partners Health Care SystemWellesley, MAUnited States; 6 VA Boston Health CareGeneral Internal MedicineBoston, MAUnited States

**Keywords:** electronic health record, physician satisfaction, implementation, Massachusetts eHealth collaborative

## Abstract

**Background:**

Despite mandates and incentives for electronic health record (EHR) adoption, little is known about factors predicting physicians’ satisfaction following EHR implementation.

**Objective:**

To measure predictors of physician satisfaction following EHR adoption.

**Methods:**

A total of 163 physicians completed a mailed survey before and after EHR implementation through a statewide pilot project in Massachusetts. Multivariable logistic regression identified predictors of physician satisfaction with their current practice situation in 2009 and generalized estimating equations accounted for clustering.

**Results:**

The response rate was 77% in 2005 and 68% in 2009. In 2005, prior to EHR adoption, 28% of physicians were very satisfied with their current practice situation compared to 25% in 2009, following EHR adoption (*P* < .001). In multivariate analysis, physician satisfaction following EHR adoption was correlated with self-reported ease of EHR implementation (adjusted odds ratio [OR] = 5.7, 95% CI 2.1 - 16), resources for practice improvement (adjusted OR = 2.6, 95% CI 1.2 - 6.1), pre-intervention satisfaction (adjusted OR = 4.8, 95% CI 1.5 - 15), and stress (adjusted OR = 5.3, 95% CI 1.1 - 25). Male physicians reported lower satisfaction following EHR adoption (adjusted OR = 0.3, 95% CI 0.2 - 0.6).

**Conclusions:**

Interventions to expand EHR use should consider additional support for practices with fewer resources for improvement and ensure ease of EHR implementation. EHR adoption may be a factor in alleviating physicians’ stress. Addressing physicians’ satisfaction prior to practice transformation and anticipating greater dissatisfaction among male physicians will be essential to retaining the physician workforce and ensuring the quality of care they deliver.

## Introduction

Electronic health records (EHRs) have the potential to transform health care by improving the quality and safety of care delivered, particularly if adopted on a wide-scale basis. EHRs may also reduce the cost of providing ambulatory care [1,2]. Despite emerging evidence about these potential benefits, increasing public policy attention toward universal EHR adoption by legislative leaders, and financial incentives for EHR use, little is known about physician experience following EHR adoption.

Successful EHR implementation generally requires users to be satisfied; therefore, defining predictors of satisfaction may increase the likelihood of a system’s success. Recent studies examining existing physician EHR users found that larger practices and younger physicians were more likely to adopt EHRs [3,4]. In addition, EHR users were found to be generally satisfied with their system [3]. Another cross-sectional study of physicians’ EHR-related satisfaction found that it was time-dependent and associated with use of a more robust EHR [5]. Prior studies have demonstrated physician satisfaction with new EHR adoption to be associated with the success of electronic implementation [6]; however, few prospective data are available regarding physicians’ overall satisfaction with their practice following EHR adoption.

It is estimated that 27% of practices in the United States were using a fully functional EHR in 2010 [7]. The mandate for universal EHR implementation and robust clinical data exchange by 2014 [8], and incentives in place for EHR adoption [7] mean that very large numbers of practices in the United States are, or likely soon will be, in the process of EHR implementation. EHR adoption may not only impact how a physician writes prescriptions and notes, but may also influence the overall practice environment by changing workflow and physician-patient interaction. In addition, the emerging patient-centered medical home as a model for health care delivery rewards practices for EHR adoption [9], even though the EHRs of today are variable at meeting the needs of medical homes [10,11]. It is likely that EHR implementation is occurring simultaneously with practice changes to fit the medical home model, further underscoring the need to assess physicians’ satisfaction with an EHR, but also with their practice overall.

Using Konrad and Linzer’s framework for measuring physicians’ work-life satisfaction [12,13], we identified several key facets that would be relevant to physicians’ satisfaction with their practice post-EHR adoption. We speculated that intrinsic factors (eg, years in practice, gender, race, self-reported stress, computer literacy, and satisfaction prior to EHR implementation), pay (in the form of bonuses for EHR use), and availability of practice resources would predict physician satisfaction following EHR adoption. Therefore, we undertook the present study to identify correlates of physician satisfaction with their practice following EHR adoption, in the context of a pilot community-based collaborative model in Massachusetts.

## Methods

The methods of survey development and administration have been described elsewhere and are summarized subsequently [14]. The survey and research protocol were approved by the Partners HealthCare Human Research Committee.

### Setting: The Massachusetts eHealth Collaborative

The Massachusetts eHealth Collaborative (MAeHC) is a not-for-profit consortium established in 2004 as a pilot demonstration project to facilitate the adoption and implementation of EHRs. Following a competitive application process involving 35 eligible communities, three communities were selected as demonstration sites in March 2005: North Adams/Williamstown, Brockton, and Newburyport. Physicians and practices within these communities were found to be generally representative of Massachusetts [14]. A total of 548 physicians (97.7%) in 159 practices (95.2%) accepted the offer to participate. A select group of EHR vendors were identified through a comprehensive process and agreed to competitive EHR pricing. The EHR vendors Allscripts, eClinicalWorks, NextGen Healthcare Information Systems, and GE Healthcare were selected (all of whom were subsequently certified by the Certification Commission for Health Information Technology). As of the pilot’s completion date [15], overall participation was 84.0% for physicians in 134 practices in the pilot’s three communities. EHRs were implemented in 97.9% of practices between March 2006 and March 2008.

### The Massachusetts eHealth Collaborative Survey

 The MAeHC physicians were surveyed using the same survey instrument developed for a Massachusetts statewide survey of EHR use, described elsewhere [4] and available from the corresponding author upon request. The survey included demographic information about the physician, including age, gender, and self-reported race (physicians had the option to select one or more of the following categories: Asian, American Indian or Alaskan Native, black or African American, Native Hawaiian or other Pacific Islander, white, or “other”). Race data were collected in order to compare survey data with census figures about population distributions. The baseline MAeHC survey was delivered to participating physicians between September and October 2005. Between January and February 2009, a similar follow-up survey was delivered after physicians had been using a new EHR for at least one year. Surveys were mailed to all physicians who had signed agreements to participate in the MAeHC. Physicians were instructed to complete the survey and return the survey by mail or to an MAeHC staff member visiting their practice site. No cash incentive was offered for survey completion because participation in the evaluations was a condition for MAeHC involvement. Between 2005 and 2009, some practices dissolved, some physicians departed, and new physicians entered the communities. A total of 163 physicians from 134 practices completed both the 2005 and 2009 survey questionnaires [16].

### Statistical Analysis

For all statistical analyses, SAS version 9.2 software (SAS Institute Inc, Cary, NC, USA) was used. Logistic regression was used to analyze predictors of self-reported physician satisfaction following EHR implementation, and Chi-square analysis was used to examine the association between physician satisfaction and perceived EHR vendor characteristics. The dependent variable in these analyses was the response to the 2009 survey item: “Overall, how satisfied are you with your current practice situation?” Physicians were asked to check 1 of 4 possible responses: very satisfied, generally satisfied, somewhat dissatisfied, or very dissatisfied. These responses were dichotomized in the analyses to “very satisfied” versus “generally satisfied, somewhat dissatisfied, or very dissatisfied.” In this logistic regression model, physician satisfaction was examined as a function of exogenous and perceptual predictor variables modeled after Konrad and Linzer’s 10 facets of measuring physician satisfaction [12,13]. Exogenous predictor variables included were whether the practice had financial incentives for EHR adoption, the level of financial resources available for practice improvement or expansion, size of practice (1-4 vs 5+ physicians or nurse practitioners and physician assistants), practice specialty (primary care vs specialty practice), and whether the practice experienced difficulty with EHR implementation (self-reported). Perceptual variables included physicians’ views of whether personal or professional stress was a problem in their practice and how comfortable they felt with the use of computers prior to EHR implementation and their daily patient volume in 2009. Physician characteristics, including age, gender, self-reported satisfaction in 2005, and practice ownership, were also included *a priori* in the final logistic regression model. Generalized estimating equations were used for the logistic regression to account for the correlations within each practice [17,18].

## Results

### Respondent Characteristics

A total of 163 surveys were returned from the same physicians in 2005 and 2009. The response rate in 2005 was 76.5% and was 68.2% in 2009. Respondents and non-respondents were similar with respect to specialty and practice size [14]. Of the 163 respondents to both surveys, 147 responded to the question, “Overall, how satisfied are you with your current practice situation?” in both the 2005 and 2009 surveys. Of the respondents to both surveys, 44 had missing responses to questions used as explanatory variables, resulting in 119 respondents’ surveys eligible for subsequent analysis.

### Physician Satisfaction

Physicians responding to both the 2005 and the 2009 MAeHC surveys had adopted an EHR for at least one year, and were primarily white (79.1%) men (73.6%) in practice for more than 15 years (89.0%). Overall, 26.4% reported feeling very satisfied with their current practice situation in 2005, compared to 23.9% in 2009 (*P* < .001). Approximately half (46.6%) of responding physicians worked in practices with > 5 physicians or nurse practitioners (NPs) or physician assistants (PAs), and 60.7% worked in specialty (non-primary care) practices. A majority (61.4%) reported incentives for health information technology (HIT) use. A total of 20.2% reported that their practices had financial resources available for practice improvement or expansion. A minority of physicians (16.6%) post-EHR adoption reported that they would prefer to give up their EHR and return to paper records (Table 1).

### Predictors of Physician Satisfaction with Current Practice

In logistic regression analyses, ease of EHR implementation and male gender were the strongest independent predictors of physician satisfaction among physicians adopting a new EHR (Table 2). Physicians who found that their EHR implementation process was not very difficult were much more likely to report feeling satisfied in their current practice in 2009 (adjusted odds ratio [OR] = 5.7, 95% CI 2.1 - 16). Male physicians were much less likely to be satisfied following EHR adoption (adjusted OR = 0.3, 95% CI 0.2 - 0.6), whereas physicians who reported higher personal or professional stress before EHR adoption were more satisfied following implementation (adjusted OR = 5.3, 95% CI 1.1 - 25). Physicians’ report of feeling very satisfied in their current practice at the time of their baseline survey in 2005 was also strongly correlated with satisfaction in 2009 (adjusted OR = 4.8, 95% CI 1.5 - 15). Physicians in practices with financial resources available for practice improvement or expansion also were more likely to report feeling satisfied in their current practice in 2009 (adjusted OR = 2.6, 95% CI 1.2 - 6.1).

In the Chi-square analysis, we found that baseline levels of satisfaction were not related to post-intervention (2009) reports of easy EHR implementation (*P* = .62). This analysis provided evidence that the observed relationship between ease of EHR implementation and 2009 satisfaction was not likely to be confounded by baseline levels of satisfaction. We also confirmed that satisfaction post-EHR adoption was strongly associated with the physician-reported training level of the EHR vendor (*P = *.002) and the quality of assistance provided by MAeHC senior leadership (*P *< .001) and staff (*P *= .002), but found that prompt support from the information technology (IT) support team for problems related to the EHR application, or for hardware or network connectivity, did not impact practice satisfaction (Table 3).

**Table 1:** Sample characteristics and demographics of physician respondents to both the 2005 and the 2009 Massachusetts eHealth Collaborative (MAeHC) surveys (n = 163).^a^

Characteristic		n (%)

**Gender**		
	Male	120 (73.6%)
	Female	38 (23.3%)
	Missing response	5 (3.1%)

**Years in practice**		
	15 or greater	145 (89.0%)
	Less than 15	18 (11.0%)

**Race**		
	White	129 (79.1%)
	Asian	15 (9.2%)
	American Indian or Alaskan Native	1 (0.6%)
	Black or African American	4 (2.5%)
	Native Hawaiian or other Pacific Islander	0
	Other	4 (2.5%)
	Missing response	10 (6.1%)

**Practice size**		
	Solo or partner practice	47 (28.8%)
	3-5 physicians or NPs/PAs	24 (14.7%)
	> 5 physicians or NPs/PAs	76 (46.6%)
	Missing response	16 (9.8%)
**Practice type** ^**b**^		
	Primary care	63 (38.7%)
	Specialty	99 (60.7%)
	Missing response	1 (0.6%)

**Moderate or extensive financial resources ** ** available for practice improvement or expansion**		33 (20.2%)

**Incentives for HIT**		100 (61.4%)

**Satisfaction with current practice**		
	Very satisfied in 2005	43 (26.4%)
	Very satisfied in 2009	39 (23.9%)

**Personal/professional stress**		
	A moderate or serious problem in 2005	57 (35.0%)
	A moderate or serious problem in 2009	61 (37.4%)

**Comfort with computers**		
	Very comfortable in 2005	74 (45.4%)
	Very comfortable in 2009	87 (53.4%)

**EHR implementation** ^**c**^		
	Very difficult	54 (33.1%)
	Somewhat or not difficult	102 (62.6%)
	Missing response	7 (4.3%)

**Preference to return to paper records in 2009** ^**d**^		
	Yes	27 (16.6%)
	No	136 (83.4%)

^a^ Not all respondents answered all questions. Responses are from 2009 survey unless otherwise indicated.

^b^ Primary care practices in 2005 include solo/group/partnership practices; specialty practices include single or multispecialty solo/group/partnership practices.

^c^ There were no responses to the answer “not sure.”

^d^ There were no responses to the answer “not applicable.”

**Table 2:** Multivariate relationship between practice characteristics and physician perceptions of practices and physician satisfaction (n = 119).^a^

Characteristic		Adjusted OR	95% CI	*P* value
**Gender**				
	Male	0.3	0.2 - 0.6	< .001
**Years in practice**				
	15 or greater	2.3	0.7 - 7.2	.17
**Race**				
	White	2.1	0.8 - 5.5	.14
**Practice ownership**		3.0	0.3 - 28	.33
**Practice size**				
	Solo or partner practice (reference)	--	--	
	3-5 physicians or NPs/PAs			
	> 5 physicians or NPs/PAs	2.5	0.8 - 7.9	.11
**Practice type** ^**b**^				
	Primary care	1.3	0.4 - 0.6	.73
**Daily patient volume**				
	≤ 15 (reference)	--	--	
	> 16	0.5	0.1 - 2.5	.40
**Moderate or extensive financial resources available for practice improvement or expansion**		2.6	1.2 - 6.1	.02
**Incentives for HIT ** ^c^		1.7	0.2 - 10	.50
**Physician perceptions**				
	Very satisfied with current practice (2005)	4.8	1.5 - 15	.007
	Personal/professional stress a moderate or serious problem (2005)	5.3	1.1 - 25	.04
	Very comfortable with computers (2005)	1.6	0.3 - 10	.60
	No difficulty with EHR implementation (2009)	5.7	2.1 - 16	< .001

^a^ Logistic regression analysis with generalized estimating equations, modeling the outcome, physician satisfaction with current practice situation, as a function of all listed characteristics. The model included all respondents (n = 163) with non-missing values for all variables included in the model.

^b^ Primary care practices in 2005 include solo/group/partnership practices.

^c^ Practice income or personal earnings for the use of electronic information systems.

**Table 3:** Bivariate relationship between physicians feeling very satisfied with their practice following EHR adoption and their perceptions of EHR vendor characteristics (n = 159).

Characteristic		*P* value
**Excellent or very good EHR vendor quality**		.002
**Excellent or very good help provided by MAeHC**		
	Senior leadership	< .001
	Senior staff	< .001
**Prompt support from information technology team**		
	For hardware/ network connectivity	.82
	For EHR application issues	.74

## Discussion

We examined physicians’ satisfaction with their practice in the context of new EHR adoption through a pilot community-based collaborative model in Massachusetts (MAeHC) and found that physician satisfaction declined modestly overall after EHR adoption. We found that one of the strongest predictors of physicians reportingsatisfaction post-EHR adoption was the ease with which the EHR implementation occurred. In subsequent analyses, we found that satisfaction was related to physician-perceived EHR vendor training and the quality of assistance provided by MAeHC senior leadership and staff. There was no association with availability of prompt support from the IT team for hardware or connectivity issues, or for problems related to the EHR application. This finding emphasizes the importance of site-specific experience and potentially explains why physicians within the MAeHC may have reported varying experiences with ease of EHR implementation.

In the context of Konrad and Linzer’s framework of the necessary facets for physician job satisfaction [13], our results support the collective presence of a practice’s resources (financial resources for improvement or expansion), community (MAeHC, a community-wide pilot project), and administrative features (ease of EHR implementation) in nurturing physician satisfaction during a period of EHR adoption. Other predictors of satisfaction included physician’s satisfaction with their practice prior to EHR implementation. We also found that increased self-reported physician stress before EHR adoption was a predictor of satisfaction following EHR implementation. Although little research exists on the issue of physician stress and workflow, our results raise the intriguing possibility that physicians with higher stress levels may perceive a greater benefit from EHR implementation, perhaps by improving workflow, efficiency, and care coordination, resulting in higher levels of satisfaction. We did not find any associations between satisfaction following EHR adoption and physician age, practice size or ownership, physicians’ patient volume, or number of years in practice. The presence of financial incentives for EHR adoption was not associated with post-adoption satisfaction in this study.

Our finding that male physicians were much less likely to report being satisfied following EHR implementation adds to the existing mixed literature on gender differences in physician career satisfaction. The largest study of female physicians, the Women Physicians’ Health Study [19], found that 84% of respondents reported being “usually,” “almost always,” or “always” satisfied. Another study demonstrated that male physicians report greater job dissatisfaction, work-life stress, and isolation in their work environment [20]. Existing social science literature has proposed several potential explanations for the higher reported satisfaction rates among professional women, including lower job expectations, socialization not to express discontent, and appreciation of different career characteristics compared with men [21-23]. The Physician Work Life Study, a large, nationally representative, randomly stratified sample, showed similar global satisfaction scores for men and women, although researchers found that women were more satisfied with their specialty and patient relationships than their levels of autonomy, relationships with the community, pay, and resources [24]. In the context of this literature, greater male dissatisfaction, particularly following practice transformation, is not an unexpected finding.

Our finding that overall physician satisfaction decreased between 2005 and 2009 may be explained by a possible ceiling effect within our study sample. We observed that 92% of physicians reported feeling very satisfied or satisfied in 2005, and 75% reported feeling very satisfied or satisfied in 2009, The physicians in our study may have been more satisfied at baseline than average physicians in Massachusetts, where the proportion of physicians reporting that they felt either very satisfied or satisfied remained approximately the same (39% in 2006 and 38% in 2009) [25]. The Massachusetts health care insurance reform law in October 2006, pressure from pay-for-performance measures, or the economic climate may have contributed toward the observed trends in our sample.

Few studies have examined physicians’ satisfaction with their practices in the context of community-based EHR adoption. One strength of this study was that EHR adoption was facilitated through the same pilot program among all surveyed physicians in three communities. This pilot was specifically designed to streamline robust EHR implementation, which has been found previously to be correlated with physician satisfaction [5]. Since participation in evaluation was a condition for MAeHC involvement, we were fortunate to have robust survey response rates for both the 2005 and 2009 surveys [15]. Surveys conducted before and after EHR implementation also allowed us to account for baseline satisfaction—our outcome of interest—in our analysis.

This study has several limitations. Despite high response rates, the sample size (n = 119) restricted the number of independent variables that could be examined as correlates of physician satisfaction. In addition, as in any observational study, unmeasured confounders may exist, although our models included a number of covariates to control for confounding to the extent possible. A 2010 study found that satisfaction with EHR use seems to increase over time, but this study was limited by a low response rate (28%) [5]. It is possible that the physicians in our study, with relatively recent EHR implementation (at least 1 year, but many less than 2 years) would have reported higher levels of overall satisfaction with their practice if surveyed at a later date [26]. Since meaningful use criteria were established after these surveys were conducted, clinician experience in the context of meaningful use and EHR implementation was not captured in this study. The study’s setting and participants, generally comparable to physicians practicing within Massachusetts [4], may have limited generalizability to other states or communities, and these results may not generalize outside the United States. In addition, because all physicians were members of practices assisted by MAeHC with EHR implementation, physicians’ experiences with EHR implementation in other communities by alternate EHR vendors may differ.

EHR adoption and meaningful use of health information exchange represent a national health care priority in the United States. Before implementation of the American Recovery and Reinvestment Act of 2009, EHR adoption rates had been sluggish in the United States. This has led policy makers to implement broad national incentives for providers, and policy makers have also actively sought successful programs as models for EHR design and implementation in order to meet the national goal of universal EHR implementation and robust clinical data exchange by 2014 [8]. Demonstration projects, such as the MAeHC, have great potential to play a transformative role in the quest for widespread EHR adoption when partnered with on-site effective leadership and staff support. Importantly, our finding of ease of EHR implementation being strongly associated with physicians’ satisfaction is particularly noteworthy because sustained usage of EHR and physician promotion of EHR use within professional networks is fundamentally dependent on physicians’ satisfaction. Despite relatively widespread use of incentives for EHR use and physicians’ reports of comfort with computer use, neither of these was found to be associated with satisfaction in practice following EHR implementation. Our data, in-line with prior literature, underscore the need to adequately support practices with fewer financial resources for improvement and ensure physician’s practice satisfaction even before embarking on EHR implementation.

## Multimedia Appendix 1

MAeHC survey 2005.

## Multimedia Appendix 2

MAeHC survey 2009.

## Abbreviations

EHR: electronic health record
HIT: health information technology
MAeHC: Massachusetts eHealth Collaborative
NP: nurse practitioner
OR: odds ratio
PA: physician assistant
