# Supplementary material for: Physician Satisfaction Following Electronic Health Record Adoption in Three Massachusetts Communities
Source: Interact J Med Res. 2012 Nov 8;1(2):e12. doi: 10.2196/ijmr.2064 (PMC3626123; doi:10.2196/ijmr.2064)
Supplement: Supplementary file 2 [file ijmr_v1i2e12_app2.pdf]

MAeHC Survey of Clinicians and Computer Technology 2009

CLINICIAN SURVEY

Instructions

This survey asks about your medical practice and factors related to the use of certain computer technology, particularly electronic health records (EHRs; also called electronic medical records). It will take about 20 minutes to complete. ***Your participation improves the quality of the results – we appreciate your help!***

All responses to the survey are private and confidential. Results will be analyzed only in the aggregate, and individual responses will not be reported in publications. Only researchers designated by MAeHC will have access to individual data collected through this survey.

Section I. Practice Characteristics

In this section, we ask you questions about your outpatient practice.

1. How long have you been associated with your current practice group? _____ ☐1 Months
_____ ☐2 Years

2. In a typical week, in how many different outpatient offices do you see patients?
   - ☐1 One
   - ☐2 Two
   - ☐3 Three or more

For the remainder of the survey, please keep in mind the office practice site where you spend the most time, your ***main*** *practice.*

---

3. Are you a
   - ☐1 Full-owner
   - ☐2 Part-owner
   - ☐3 Not an owner of the practice

4. Considering all full- and part-time clinicians at your main practice, including yourself, how many are:

   Physicians (MD or DO) _______

   Nurse Practitioners or Physician Assistants _______

5. Please **estimate** the number of outpatient visits **you** yourself had in a **typical week before implementing your EHR.**

   outpatient visits _______

6. How many hours per week do you see patients at your office? hours _______

7. How many patients do you see per day (average)? patients _______

8. Thinking of your main practice, please indicate how much of a problem each of the following is for you: *(Please check only **one** box per row.)*

	Not a Problem	Slight Problem	Moderate Problem	Serious Problem
a) Isolation from colleagues	☐1	☐2	☐3	☐4
b) Personal or professional stress	☐1	☐2	☐3	☐4
c) Having to work long hours to meet practice demands	☐1	☐2	☐3	☐4
d) Feeling demoralized about the state of medical practice in general	☐1	☐2	☐3	☐4

9. Please indicate your agreement or disagreement with the following statements, considering your main office practice:

	Strongly Agree	Agree	Neither Agree nor Disagree	Disagree	Strongly Disagree
a) The office staff are innovative	☐1	☐2	☐3	☐4	☐5
b) The physician(s) are innovative	☐1	☐2	☐3	☐4	☐5
c) Among my colleagues, I am usually one of the first to find out about a new diagnostic test or treatment	☐1	☐2	☐3	☐4	☐5
d) We are actively doing things to improve quality of care	☐1	☐2	☐3	☐4	☐5
e) After we make changes to improve quality, we evaluate their effectiveness	☐1	☐2	☐3	☐4	☐5
f) We have quality problems in our practice	☐1	☐2	☐3	☐4	☐5
g) Our procedures and systems are good at preventing errors from occurring	☐1	☐2	☐3	☐4	☐5

10. Overall, how satisfied are you with your current practice situation? *(Check one)*

☐1 Very satisfied
☐2 Generally satisfied
☐3 Somewhat *dis*satisfied
☐4 Very *dis*satisfied

Section II: Health Information Technology

The next set of questions asks about use of computer technology in your main office practice.

11. How comfortable are you with computers?
    - ☐1 Very comfortable
    - ☐2 Somewhat comfortable
    - ☐3 Not very comfortable
    - ☐4 Not at all comfortable

12. For each outcome listed below, indicate whether you think the effect of computers is, or would be, very positive, somewhat positive, no effect, somewhat negative, or very negative:

Effect of computers on…	Very Positive	Somewhat Positive	No Effect	Somewhat Negative	Very Negative
a) Controlling costs of health care	☐1	☐2	☐3	☐4	☐5
b) Quality of health care	☐1	☐2	☐3	☐4	☐5
c) Interactions within the health care team	☐1	☐2	☐3	☐4	☐5
d) Workflow	☐1	☐2	☐3	☐4	☐5
e) Patient-physician communication	☐1	☐2	☐3	☐4	☐5
f) Patient privacy	☐1	☐2	☐3	☐4	☐5
g) Clinicians' access to up-to-date knowledge	☐1	☐2	☐3	☐4	☐5
h) Efficiency of providing care	☐1	☐2	☐3	☐4	☐5
i) Medication errors	☐1	☐2	☐3	☐4	☐5

13. With your current medical record system (paper and/or electronic), how easy would it be for you or your staff to generate the following information about your patients?

	Very Easy	Somewhat Easy	Somewhat Difficult	Very Difficult	Cannot Generate
a) List of patients by diagnosis or health risk (e.g., diabetes)	☐1	☐2	☐3	☐4	☐5
b) List of patients by laboratory results (e.g., patients with abnormal hematocrit levels)	☐1	☐2	☐3	☐4	☐5
c) List of patients by medications they currently take (e.g., patients on warfarin)	☐1	☐2	☐3	☐4	☐5

14. For each task, please tell us whether you perform it on patients with the specific disease condition **at least once a year** by either paper or electronic means.

Do this at least once a year for patients with: ⇨	i) Diabetes	ii) Coronary Artery Disease
a) Prompt your practice to notify patients who are overdue for office visits	☐1 Yes ☐2 No	☐1 Yes ☐2 No
b) Generate a list of patients overdue for tests	☐1 Yes ☐2 No	☐1 Yes ☐2 No
c) Generate a list of patients with clinical data suggesting that they need an intervention (e.g. HbA1c > 7, Systolic BP > 140, etc.)	☐1 Yes ☐2 No	☐1 Yes ☐2 No

15. Upon completing a typical office visit, how do you generate medication prescriptions? (*Check all that apply*)

☐1 Computerized, using available decision support (e.g. drug interaction/allergy alerts)
☐2 Computerized, do not use available decision support
☐3 Handwritten
☐4 Other (Describe:____)

16. Please indicate all **features of the EHR** that you **have available** in your practice. For those features that you have, indicate the extent to which **you use** them:

	Available				Use		
**Features of your EHR**	Yes	No	Don't Know		I do not use	I use *some* of the time	I use *most or all* of the time
a) Laboratory test results	☐1	☐2	☐3	→	☐1	☐2	☐3
b) Laboratory order entry	☐1	☐2	☐3	→	☐1	☐2	☐3
c) Radiology test results	☐1	☐2	☐3	→	☐1	☐2	☐3
d) Radiology order entry	☐1	☐2	☐3	→	☐1	☐2	☐3
e) Electronic visit notes	☐1	☐2	☐3	→	☐1	☐2	☐3
f) Reminders for care activities (e.g. overdue health maintenance)	☐1	☐2	☐3	→	☐1	☐2	☐3
g) Electronic medication lists of what each patient takes	☐1	☐2	☐3	→	☐1	☐2	☐3
h) Electronic problem list	☐1	☐2	☐3	→	☐1	☐2	☐3
i) Can transmit prescriptions to pharmacy electronically or via FAX	☐1	☐2	☐3	→	☐1	☐2	☐3
j) Electronic referrals or clinical messaging (secure e-mailing between providers)	☐1	☐2	☐3	→	☐1	☐2	☐3

17. With what proportion of your patients do you communicate by email? *(Check one)*

- ☐1 None
- ☐2 Some, but less than 10%
- ☐3 10% or more

18. If given the choice, would you prefer to give up your EHR and go back to paper records?

- ☐1 Yes
- ☐2 No
- ☐3 Not sure

19. Before implementing your EHR, how many times did you have difficulty accessing patient records in a typical month? (e.g. difficulty finding patient charts, missing paperwork, etc.)

- ☐1 Zero
- ☐2 1-2 times
- ☐3 3-4 times
- ☐4 5 or more times

20. Since implementing your EHR, how many times do you have difficulty accessing patient records in a typical month?

- ☐1 Zero
- ☐2 1-2 times
- ☐3 3-4 times
- ☐4 5 or more times

21. How much do you agree with the following statements:

	Strongly agree	Agree	Neither agree nor disagree	Disagree	Strongly disagree
a) Implementing the EHR has hurt my earning potential from clinical activities	☐1	☐2	☐3	☐4	☐5
b) Implementing the EHR has been more costly than we originally anticipated	☐1	☐2	☐3	☐4	☐5
c) Using the EHR has created new opportunities for errors	☐1	☐2	☐3	☐4	☐5
d) Our EHR has created more errors than it has prevented	☐1	☐2	☐3	☐4	☐5
e) I feel I have less control over my practice now that I have implemented the EHR	☐1	☐2	☐3	☐4	☐5
f) Overall, the EHR has met my expectations.	☐1	☐2	☐3	☐4	☐5
g) The EHR has helped to streamline processes and increase office productivity.	☐1	☐2	☐3	☐4	☐5

22. How much of a barrier is each of the following to beginning or expanding the use of computer technology in your main practice?

	Not a barrier	Minor barrier	Major barrier
a) Computer skills of you and/or colleagues/staff	☐1	☐2	☐3
b) Computer technical support	☐1	☐2	☐3
c) Lack of time to acquire knowledge about systems	☐1	☐2	☐3
d) Start-up financial costs	☐1	☐2	☐3
e) Ongoing financial costs	☐1	☐2	☐3
f) Training and productivity loss	☐1	☐2	☐3
g) Physician skepticism	☐1	☐2	☐3
h) Privacy or security concerns	☐1	☐2	☐3
i) Lack of uniform standards within industry (e.g., having to use multiple systems used by different providers and plans)	☐1	☐2	☐3
j) Technical limitations of systems	☐1	☐2	☐3
k) Staff skepticism	☐1	☐2	☐3
l) Workflow adjustments	☐1	☐2	☐3

The next few questions are about health information exchange, or HIE. For the purposes of answering these questions, our **definition of HIE is the ability for clinicians to share a core set of clinical patient data across practices and entities**. For example, a patient whose PCP is in Holyoke, MA, goes to Springfield to the ER – her medication list, allergies and laboratory results would be retrievable in the Springfield ER department if her PCP office and the hospital have both implemented HIE.

The ability to share patient data within an office group or practice does ***not*** qualify as HIE, ***nor*** does the ability to fax/email/mail patient information to other entities.

23. What effect do you think health information exchange (HIE) would have in relation to the areas below:

	Very Positive	Somewhat Positive	No Effect	Somewhat Negative	Very Negative
a) Reducing health care costs	☐1	☐2	☐3	☐4	☐5
b) Improving quality of patient care	☐1	☐2	☐3	☐4	☐5
c) Saving time for clinicians	☐1	☐2	☐3	☐4	☐5

24. To what degree are you concerned with privacy and security as related to HIE?

- ☐1 Very concerned
- ☐2 Somewhat concerned
- ☐3 Not at all concerned

25. How important are the following HIE functions to you:

	Very Important	Somewhat Important	Not Important
a) Problem list	☐1	☐2	☐3
b) Procedure history	☐1	☐2	☐3
c) Medication list	☐1	☐2	☐3
d) Allergies	☐1	☐2	☐3
e) Consultation tracking	☐1	☐2	☐3
f) Patient demographics	☐1	☐2	☐3
g) Patient portal	☐1	☐2	☐3

26. To what degree are you enthusiastic about the prospect of being able to exchange patient data via HIE?

- ☐1 Very enthusiastic
- ☐2 Somewhat enthusiastic
- ☐3 Not at all enthusiastic

27. If participation in HIE were like a cable bill with a monthly charge, would you consider participating?

- ☐1 Yes
- ☐2 No

28. What is the most you would pay each month per clinician to participate in HIE?

- ☐1 $25-50
- ☐2 $51-100
- ☐3 $101-150
- ☐4 Would not pay

29. For what percent of outpatient visits do you check the HIE for records?

___ ___ ___ % ☐1 Check here if your HIE is not operational

30. Of the visits you check the HIE for records, for what percentage do you find the patients you are looking for?

___ ___ ___ % ☐1 Check here if your HIE is not operational

31. For what percent of those patients that you locate in the HIE do you find useful patient information?

___ ___ ___ % ☐1 Check here if your HIE is not operational

Section III: Financial Considerations

The following five questions concern your practice's financial profile.

32. Please indicate below whether the following factors (through bonuses, returned withholds, or other incentive payments) contribute to **either** your **practice's income**, or **your personal earnings**?

	**Practice's Income** Yes	**Practice's Income** No	**Personal Earnings** Yes	**Personal Earnings** No
a) Types of electronic information systems you **have** (e.g., EHRs, e-prescribing)	☐1	☐2	☐1	☐2
b) The amount you **use** electronic information systems	☐1	☐2	☐1	☐2
c) Patient survey results (e.g. satisfaction)	☐1	☐2	☐1	☐2
d) Clinical quality (e.g., "pay for performance")	☐1	☐2	☐1	☐2

33. Approximately what percent of your 2008 **clinical practice income** was earned in the form of bonuses, returned withholds, or other incentive payments **based on the use of electronic health record systems or electronic prescribing**?

- ☐1 0% of income
- ☐2 1 - 5% of income
- ☐3 6 - 10% of income
- ☐4 more than 10% of income
- ☐5 Not sure

34. Practices vary with respect to the capital they have available for expansion and improvement. What financial resources does your main practice have for expansion or improvements of any kind?
   - ☐1 Extensive resources
   - ☐2 Moderate resources
   - ☐3 Limited resources
   - ☐4 No resources

35. Since implementing the EHR, have your coding levels:
   - ☐1 Increased
   - ☐2 Decreased
   - ☐3 No significant difference in levels

36. Have your coding patterns changed in any other way since implementing the EHR?
   - ☐1 Yes
   - ☐2 No
   - ☐3 Not sure

Section IV: Patient Flow and Documentation Analysis

In this section, we ask you about your office workflow.

37. What **method** do you currently use to document patient encounters?
   - ☐1 Handwritten
   - ☐2 Dictated and transcribed
   - ☐3 Voice recognition
   - ☐4 Entered directly into EHR
   - ☐5 Combination, please specify rough proportions: ____________________________
     ____

38. **When** do you document patient encounters?
   - ☐1 During the encounter
   - ☐2 At the end of the encounter
   - ☐3 Lunch/end of day
   - ☐4 Other: ________

39. **Where** do you document patient encounters?
   - ☐1 In exam room with patient
   - ☐2 In the office
   - ☐3 At nurse/dictation station
   - ☐4 Other: _________

40. How do you document the following?

	EHR	Paper	N/A
a) Medications	☐1	☐2	☐3
b) Allergies	☐1	☐2	☐3
c) History	☐1	☐2	☐3
d) HPI	☐1	☐2	☐3
e) Immunizations	☐1	☐2	☐3
f) Office-based encounters	☐1	☐2	☐3
g) Out-of-office encounters	☐1	☐2	☐3
h) Virtual visit encounters	☐1	☐2	☐3

41. How frequently do you use the following?

	Almost always	Usually	Sometimes	Never/hardly ever
a) Templates	☐1	☐2	☐3	☐4
b) Flowsheets	☐1	☐2	☐3	☐4
c) Forms designed to be scanned into the EHR (e.g. bubblesheets)	☐1	☐2	☐3	☐4
d) Medication lists	☐1	☐2	☐3	☐4
e) Problem lists	☐1	☐2	☐3	☐4
f) Medical alerts	☐1	☐2	☐3	☐4
g) Billing alerts	☐1	☐2	☐3	☐4
h) Patient recalls	☐1	☐2	☐3	☐4
i) Prescription refills	☐1	☐2	☐3	☐4
j) Letters	☐1	☐2	☐3	☐4
k) Voice recognition/dictation	☐1	☐2	☐3	☐4
l) Note locking/signing/closing	☐1	☐2	☐3	☐4

Section V: Implementation Process

The next few questions ask you about your experience with EHR implementation.

42. Was the implementation process for your EHR:
- ☐1 Very difficult
- ☐2 Somewhat difficult
- ☐3 Not difficult
- ☐4 Not Applicable

43. Please rate the following:

	Excellent	Very good	Fair	Poor	Not Applicable
a) EHR *vendor's* training	☐1	☐2	☐3	☐4	☐5
b) Help provided by MAeHC *Practice Consultant*	☐1	☐2	☐3	☐4	☐5
c) Help provided by MAeHC *Senior Pilot Executive*	☐1	☐2	☐3	☐4	☐5
d) Help provided by *other* MAeHC staff	☐1	☐2	☐3	☐4	☐5

44. How much do you agree with the following statements?

	Strongly agree	Somewhat agree	Somewhat *dis*agree	Strongly *dis*agree
a) You receive prompt IT (*hardware, network connectivity, etc.*) support when required.	☐1	☐2	☐3	☐4
b) You are satisfied with the resolutions to your IT issues.	☐1	☐2	☐3	☐4
c) You receive prompt application (*EHR*) support when required.	☐1	☐2	☐3	☐4
d) You are satisfied with the resolutions to your application issues.	☐1	☐2	☐3	☐4

Section VI: Personal Characteristics

We would like to end this survey by asking about general background information that may help us interpret survey findings and determine how representative our sample is.

45. What is your medical specialty? ____
Or ☐ check here if you are a PA or NP

46. What is your medical subspecialty? ____________________________________
Or ☐ check here if none

Please go on to back page …

47. What type of clinician are you?

- ☐1 MD
- ☐2 DO
- ☐3 NP – Nurse Practitioner
- ☐4 PA – Physician Assistant
- ☐5 Other, please specify ___________________

48. In what year did you finish your professional degree (MD, DO, PhD, DPM, NP, etc.):
______ year graduated

49. In what year were you born? _______

50. Are you

- ☐1 Male
- ☐2 Female

51. Are you of Hispanic or Latino origin?

- ☐1 Yes
- ☐2 No

52. Please answer this question whether or not you are Hispanic or Latino. This allows us to compare data with census figures about population distributions. What is your race? **Select one or more** of the following -

- ☐1 Asian
- ☐2 American Indian or Alaska Native
- ☐3 Black or African American
- ☐4 Native Hawaiian or Other Pacific Islander
- ☐5 White
- ☐6 Other

53. Date survey completed: ___ ___ / ___ ___ / ___ ___ ___ ___

<u>Thank you</u> for completing this survey!

We greatly appreciate your participation.

Please return your completed survey in the enclosed, postage-paid envelope.
